# Robustness and dosimetric verification of hippocampal-sparing craniospinal pencil beam scanning proton plans for pediatric medulloblastoma

**DOI:** 10.1016/j.phro.2024.100555

**Published:** 2024-02-15

**Authors:** Anneli Edvardsson, Jenny Gorgisyan, Karin M. Andersson, Christina Vallhagen Dahlgren, Alexandru Dasu, Daniel Gram, Thomas Björk-Eriksson, Per Munck af Rosenschöld

**Affiliations:** a Radiation Physics, Department of Hematology, Oncology and Radiation Physics, Skåne University Hospital, Sweden; b Medical Radiation Physics, Department of Clinical Sciences Lund, Lund University, Lund, Sweden; c The Skandion Clinic, Uppsala, Sweden; d Medical Radiation Sciences, Department of Immunology, Genetics and Pathology, Uppsala University, Uppsala, Sweden; e Department of Clinical Oncology and Palliative Care, Radiotherapy, Zealand University Hospital, Næstved, Denmark; f Niels Bohr Institute, University of Copenhagen, Copenhagen, Denmark; g Department of Oncology – Section of Radiotherapy, Rigshospitalet, Copenhagen, Denmark; h Department of Oncology, Institute of Clinical Sciences, Sahlgrenska Academy at the University of Gothenburg, Gothenburg, Sweden; i Regional Cancer Centre West, Western Sweden Healthcare Region, Gothenburg, Sweden

**Keywords:** Pediatrics, Medulloblastoma, Craniospinal irradiation, Proton therapy, Hippocampal-sparing, Quality assurance

## Abstract

**Background and Purpose:**

Hippocampal-sparing (HS) is a method that can potentially reduce late cognitive complications for pediatric medulloblastoma (MB) patients treated with craniospinal proton therapy (PT). The aim of this study was to investigate robustness and dosimetric plan verification of pencil beam scanning HS PT.

**Materials and Methods:**

HS and non-HS PT plans for the whole brain part of craniospinal treatment were created for 15 pediatric MB patients. A robust evaluation of the plans was performed. Plans were recalculated in a water phantom and measured field-by-field using an ion chamber detector at depths corresponding to the central part of hippocampi. All HS and non-HS fields were measured with the standard resolution of the detector and in addition 16 HS fields were measured with high resolution. Measured and planned dose distributions were compared using gamma evaluation.

**Results:**

The median mean hippocampus dose was reduced from 22.9 Gy (RBE) to 8.9 Gy (RBE), while keeping CTV V_95%_ above 95 % for all nominal HS plans. HS plans were relatively robust regarding hippocampus mean dose, however, less robust regarding target coverage and maximum dose compared to non-HS plans. For standard resolution measurements, median pass rates were 99.7 % for HS and 99.5 % for non-HS plans (p < 0.001). For high-resolution measurements, median pass rates were 100 % in the hippocampus region and 98.2 % in the surrounding region.

**Conclusions:**

A substantial reduction of dose in the hippocampus region appeared feasible. Dosimetric accuracy of HS plans was comparable to non-HS plans and agreed well with planned dose distribution in the hippocampus region.

## Highlights

• Reduced hippocampus dose from 23 to 9 Gy (RBE) with acceptable target coverage.
• Hippocampal-sparing plans were robust with respect to hippocampus mean dose.
• Dosimetric accuracy was within 3 % of the planned dose for hippocampal-sparing plans.
• Comparable dosimetric accuracy between hippocampal-sparing and conventional plans.

## 1 Introduction

Medulloblastoma (MB) is the most common primary malignant brain tumor in children [1]. For patients above 3 years of age, MB are generally treated with a combination of surgery, chemotherapy and radiotherapy (RT) [1]. Because of a high risk of dissemination along the neural axis, MB is treated with postoperative craniospinal irradiation (CSI) together with a boost to the tumor bed. Treatment depends on various risk factors, such as residual tumor volume, M−stage, histology and molecular subgroup [2], [3]. Survival has improved greatly over the last decades [4] and currently the 5-year survival is above 80 % for standard-risk patients [5], [6]. However, many survivors experience severe late side-effects, e.g., cognitive impairment, loss of hearing/vision, hypothyroidism, loss of gonadal function and even fatal heart and lung complications [2], [7]. Introducing proton therapy (PT) CSI, the dose to many organs-at-risk (OAR) is decreased compared to photon CSI, reducing the risk of late side-effects [7], [8]. Thus, PT has shown superior cognitive outcomes compared to conventional photon treatment for pediatric MB patients, however, there is still an increased risk of late cognitive complications [9], [10], [11].

Several studies have shown an association between radiation dose to hippocampus and late cognitive complications in pediatric brain tumor patients [12], [13], [14], [15], [16]. Within the subgranular zone of the hippocampal dentate gyrus neurogenesis takes place, a process that occurs throughout life [17], [18], [19]. It has been hypothesized that alteration of hippocampal neurogenesis plays an essential role in radiation-induced late cognitive complications [17], [18]. Reducing CSI dose for standard-risk MB patients from 23.4 to 18 Gy resulted in inferior event free survival rates in the dose reducing arm [5]. However, hippocampal-sparing (HS) whole brain (WB) RT with intensity modulated RT has been shown to better preserve cognitive function for adult patients with brain metastases [20]. Consequently, HS RT can bring great benefits to pediatric patients that are more susceptible to develop late cognitive complications [17], [21], [22], [23], [24], [25], [26], [27]. However, no clinical trials of HS RT for pediatric patients have yet been performed or published to our knowledge.

Simulation studies have shown that it is possible to reduce the hippocampus dose while maintaining what is usually considered clinically acceptable coverage of the clinical target volume (CTV) for pediatric MB patients [22], [23], [24], [25], [26], [27]. With lower doses to hippocampi we have previously estimated a decreased risk of late cognitive complications [24], [25], [26], with largest benefits for PT compared to various photon treatment techniques [22], [24], [25], [26], [27]. To achieve a homogeneous dose to the rest of the brain while sparing hippocampus, treatment plans with steep dose gradients are required. Such gradients could be achieved with protons due to their sharp dose fall-off at the end of the beam [25].

Safety, efficacy and toxicity of HS PT for pediatric MB patients should be evaluated in a prospective clinical trial. Before that, it remains to ensure that the planned dose distribution of this novel and very complex treatment technique can be accurately delivered to the patient. Gram et al., [25] have previously developed a treatment planning strategy for HS PT. The aim of this study was to explore robustness and dosimetric plan verification for this strategy.

## 2 Material and methods

### 2.1 Patient characteristics

Fifteen pediatric MB patients were included in this retrospective study. The patients had previously been treated with either photon or proton CSI treatment at Skåne University Hospital or at the Skandion Clinic in Sweden during 2013–2022. Characteristics of the patient cohort are presented in supplementary table 1. The study was approved by the Swedish ethical review authority (Dnr 2023–04739-01).

### 2.2 Imaging and contouring

All patients were immobilized in supine position and computed tomography (CT)- and magnetic resonance (MR) scanned headfirst supine. MR-scans included T1 with contrast and FLAIR. The original elective WB clinical target volume (CTV_WB_) and OAR structures, delineated for clinical treatment based on CT and MR images, were used in this study. Spinal part of the target was disregarded as primary interest was dose to the hippocampus area. OARs considered were brainstem, chiasm, cochlea, hippocampus, lenses, optic nerves, and pituitary gland. No boost plans were accounted for in this study since we assumed that an HS approach would not affect QA measurement results of the boost plan.

### 2.3 Treatment planning

Assuming a constant relative biological effectiveness (RBE) of 1.1, all cases were prescribed 23.4 Gy (RBE) in 13 fractions, irrespective of their original dose prescription. Proton pencil beam scanning (PBS) plans were created in Eclipse™ treatment planning system (TPS, Varian medical systems, Palo Alto, CA, USA). One posterior and two lateral fields were used in each case (gantry angles 90, 180 and 270°). Plans were created using multi-field optimization (MFO), field-specific targets margins of 3.5 % and 5 mm, and 3 mm spot spacing. Plans were robustly optimized using the Nonlinear Universal Proton Optimizer (NUPO, version 15.6.03) and the dose was calculated with the Proton Convolution Superposition algorithm (PCS version 15.6.04). A range shifter (RS) corresponding to a water equivalent thickness of 3.5 cm was used if necessary to achieve adequate dose coverage superficially (no RS - eight patients; 180° field only - four patients; all fields - three patients).

**Fig. 1:**
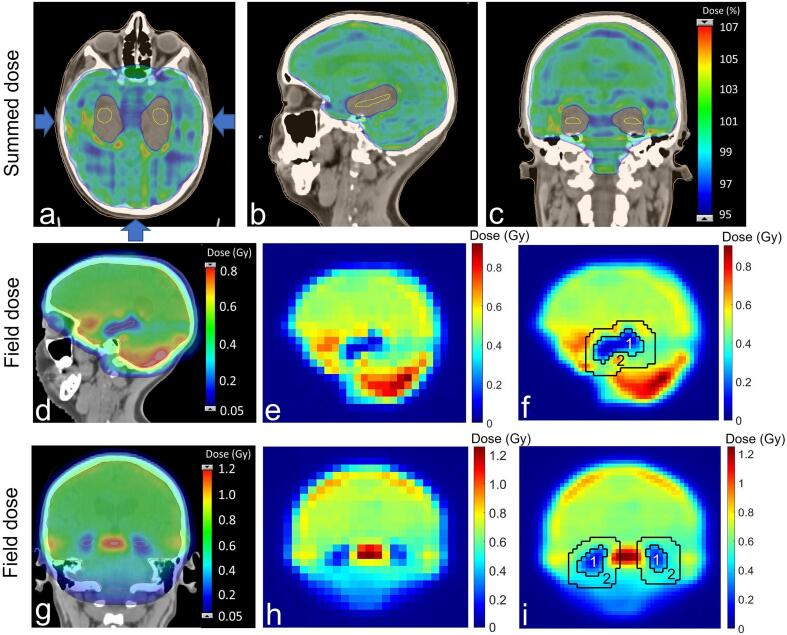
Calculated summed dose distributions for the cranial fields of a hippocampal-sparing plan (a-c) for one of the patients. Field directions are shown by arrows in a. Planned field dose distributions (d, g) and corresponding measured 2D dose distributions in standard resolution (e, h) and high resolution (f, i), with regions of interest corresponding to hippocampus (1) and surrounding (2) regions.

For each patient, one HS and one non-HS plan (Fig. 1) were generated, based on the treatment planning strategy developed by Gram et al., [25]. Non-HS plans had CTV_WB_ coverage of 95–107 % of the prescribed dose. Dose to lenses was kept to a minimum, hotspots in the rest of the OARs were avoided, and maximum dose in the body structure was minimized. Non-HS plans were robustly optimized to CTV_WB_ using 2%/2mm perturbations based on the results of Gram et al., [25]. HS plans were optimized with an additional objective to lower the mean dose to hippocampus to 9 Gy (RBE). The choice of 9 Gy (RBE) is based on the results of Gram et al., [25], which showed that for a mean hippocampus dose of 9 Gy (RBE) and 2%/2mm perturbations all plans were deemed clinically acceptable regarding target coverage. Treatment plans were robustly optimized on hippocampi and a structure corresponding to CTV_WB_ minus hippocampus with a 2 mm margin (CTV_WB,HS_) using 2%/2mm perturbations. Additional structures were also created to help the optimizer to generate sharp dose gradients around hippocampus to cover as much of CTV_WB,HS_ as possible with 95–107 % of the prescribed dose. Same objectives were used for all patients, only minor changes were made when needed to achieve adequate plan quality. Finally, both HS and non-HS plans were robustly evaluated in Eclipse using 2%/2mm perturbations.

### 2.4 Dose volume histogram analysis

**Fig. 2:**
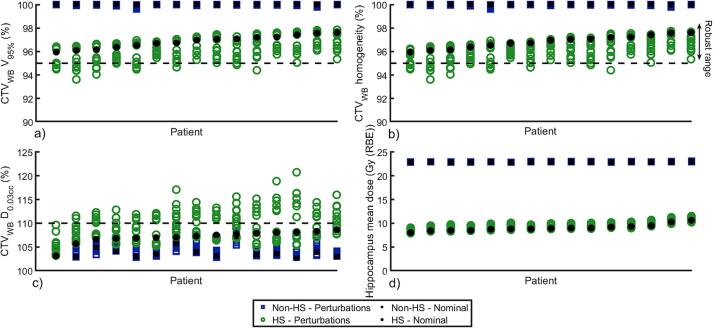
Results of the robust evaluation (2%/2mm perturbations) for hippocampal-sparing (HS, green rings) and non-hippocampal-sparing (non-HS, blue squares) plans together with nominal values (filled black markers) for a) CTV_WB_ V_95%_ (%), b) CTV_WB_ homogeneity (%), c) CTV_WB_ D_0.03cc_ (%) and d) hippocampus mean dose (Gy (RBE)). The dashed lines in (a) and (b) represent 95 % and in (c) 110 % of the prescribed dose (criteria for clinically acceptable plans). Patients are sorted in ascending order based on nominal value for the HS plan. Robust range is defined as the range of each parameter for all perturbed dose distributions (marked with an arrow in b). (For interpretation of the references to colour in this figure legend, the reader is referred to the web version of this article.)

For both nominal and perturbed (2%/2mm) dose distributions, the percentage of CTV_WB_ receiving ≥ 95 % (V_95%_) and ≤ 107 % (V_107%_) of prescription dose, as well as the dose received by 0.03 cc (D_0.03cc_) of CTV_WB_ were retrieved. Homogeneity was calculated as the relative volume of CTV_WB_ that received 95–107 % of prescribed dose. Also, mean hippocampus dose was retrieved. The range of the robust evaluation (robust range) was calculated for each parameter as the range for all perturbed dose distributions (Fig. 2).

### 2.5 Measurements

Treatment plans were delivered at the Skandion Clinic, which uses a gantry-based Proteus Plus proton therapy system (IBA, Louvain-la-Neuve, Belgium), with dedicated scanning nozzles delivering beam energies from 60 to 226 MeV. Treatment fields reset to gantry angle 0° were first recalculated in a cubic water phantom in the TPS and then measured field-by-field at the same gantry angle using the two-dimensional ion chamber array detector MatriXX PT (IBA, Schwarzenbruck, Germany) and solid water blocks as buildup material. Measurements were performed with the ion chamber array positioned perpendicular to the beam direction. Measurements were performed at water equivalent depths (WED) corresponding to the central part of each hippocampus, approximately 5 and 11 cm for lateral fields and 10 cm for posterior fields. For all patients, both HS and non-HS plans were measured with standard resolution of the detector (7.6 mm). In total, 150 standard resolution measurements were performed. Measured and planned dose distributions were compared using 2D global gamma evaluation (3%/2mm, threshold 5 %) in the myQA software (IBA Dosimetry) and resulting pass rates were compared between HS and non-HS plans. To obtain more detailed dosimetric evaluation of the hippocampus region, 16 fields for five HS plans were also measured with high resolution (3.8 mm). This was achieved by shifting the detector, resulting in 4 times more measurements compared to standard resolution. To make the high-resolution measurements representative for the whole cohort, two of the HS plans with relatively low pass rate in the standard resolution measurements and three of the HS plans with relatively high pass rate were randomly selected. For the high-resolution measurements, a 2D global gamma evaluation (3%/1mm, threshold 5 %) was performed within regions of interest (ROIs) corresponding to hippocampus and surrounding regions (Fig. 1).

### 2.6 Statistical analysis

Analyses were performed in Matlab version 2021b (MathWorks Inc., Natick, MA, USA). Nominal treatment planning parameters, robust range and pass rates for standard resolution measurements were not normally distributed according to one-sample Kolmogorov-Smirnov tests. Two-sided paired Wilcoxon tests were therefore carried out to evaluate differences in these parameters between HS and non-HS treatment. Associations between pass rates for standard resolution measurements and patient age, CTV_WB_ volume, hippocampus volume, use of RS and measurement WED for both HS and non-HS measurements were investigated using Spearman’s rank correlation. Values of p < 0.05 were considered statistically significant.

## 3 Results

### 3.1 Dose volume histogram analysis

Both nominal plan and robust evaluation parameters are presented in Fig. 2. For nominal plans, median (range) mean hippocampus dose was reduced from 22.9 Gy (RBE) (22.8–23.0 Gy (RBE)) for non-HS treatment to 8.9 Gy (RBE) (8.0–10.6 Gy (RBE)) for HS treatment (p < 0.001). Both median CTV_WB_ V_95%_ and homogeneity were 97.0 % (96.0–97.6 %) for HS treatment and 100 % (100–100 %) for non-HS treatment (p < 0.001). CTV_WB_ D_0.03cc_ was 107.3 % (103.1–108.6 %) for HS and 103.5 % (102.6–106.8 %) for non-HS treatment (p < 0.001).

For HS and non-HS plans, median (range) robust range were 2.4 % (1.4–3.1 %) and 0.1 % (0.0–0.4 %) (p < 0.001) for CTV_WB_ V_95%_, 2.2 % (1.2–3.1 %) and 0.1 % (0.0–0.4 %) (p < 0.001) for CTV_WB_ homogeneity, and 7.4 % (5.2–15.4 %) and 1.6 % (1.0–5.4 %) (p < 0.001) for CTV_WB_ D_0.03cc_ (Fig. 2). Median (range) robust range for hippocampus mean dose were 1.2 Gy (RBE) (1.0–1.5 Gy (RBE)) for HS plans and 0.1 Gy (RBE) (0.1–0.2 Gy (RBE)) for non-HS plans (p < 0.001) (Fig. 2). In the perturbed dose distributions, both V_95%_ and homogeneity dropped just below 95 % for 8/15 patients, and D_0.003cc_ was above 110 % for 14/15 patients with a maximum value of 121 % (Fig. 2). Hot spots were primarily located around the HS volume.

### 3.2 Measurements

**Fig. 3:**
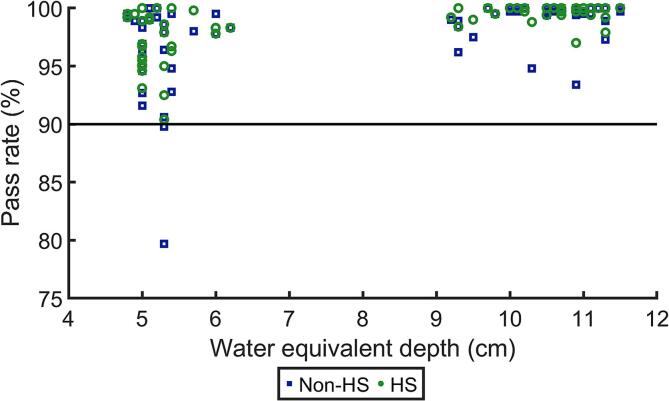
Gamma pass rates (3%/2mm, global) for standard resolution measurements comparing hippocampal-sparing plans (HS, green rings) and non-hippocampal-sparing (non-HS, blue squares). (For interpretation of the references to colour in this figure legend, the reader is referred to the web version of this article.)

Example of 2D dose distributions for standard and high-resolution measurements are presented in Fig. 1. For standard resolution measurements, median (range) pass rates were 99.7 % (90.4–100 %) for HS treatment and 99.5 % (79.7–100 %) non-HS treatment (p < 0.001) (Fig. 3). Results of the Spearman correlation are presented in Table 1. Pass rate for standard resolution measurements and measurement WED were strongly positively correlated (r_s_ = 0.68, p < 0.001), and pass rate for standard resolution measurements and CTV_WB_ volume were negatively weakly correlated (r_s_ = -0.26, p = 0.03). For high-resolution measurements, median (range) pass rates were 100 % (91.1–100 %) in the hippocampus region and 98.2 % (78.0–100 %) in the surrounding region.

**Table 1:** Spearman correlation coefficients of the pass rate for standard resolution measurements and various treatment characteristics. Statistically significant correlation (p < 0.05) is marked in bold.

	Pass rate			
	HS		Non-HS	
	r_s_	p	r_s_	p
Age (y)	−0.01	0.95	0.05	0.68
CTV_WB_ volume (cm^3^)	**−0.26**	**0.02**	**−0.25**	**0.03**
Hippocampus volume (cm^3^)	0.18	0.11	0.18	0.12
Range shifter	0.00	0.98	−0.17	0.14
Measurement WED (cm)	**0.55**	**<0.001**	**0.58**	**<0.001**

Abbreviations: r_s_ = Spearman’s rank correlation coefficient, HS = hippocampal-sparing, WED = water equivalent distance.

## 4 Discussion

To our knowledge, this is the first comprehensive study evaluating the robustness and dosimetric plan verification of HS proton PBS treatment. Results showed that it was possible to reduce mean hippocampus dose from 23 to 9 Gy (RBE), while keeping CTV_WB_ V_95%_ and homogeneity above 95 % as well as D_0.03cc_ below 110 % in the nominal plans. HS plans were relatively robust with respect to hippocampus mean dose, however, less robust regarding target coverage and maximum dose compared to non-HS plans. QA measurement results for HS plans were comparable to non-HS plans and measurements showed good agreement in the hippocampus region with dosimetric accuracy within 3 % of planning.

Previous simulation studies have shown that it is possible to create HS PBS proton plans for CSI of pediatric MB patients [22], [24], [25], [26], [27], and that these plans are estimated to reduce the risk of late cognitive complications [24], [25], [26]. Aljabab et al., [22] investigated hypothalamic-pituitary axis and hippocampus avoidance using PT for pediatric standard-risk MB patients. They showed that it was possible to lower hippocampus mean dose from 23.8 to 14.7 Gy (RBE) for the CSI (boost excluded) while keeping mean CTV D_95%_ at 97.3 %. They performed 2D QA measurements for one of the plans using the Matrixx PT detector showing a Gamma pass rate > 90 % for 3%/3mm. Blomstrand et al., [24] showed that HS PBS PT for pediatric MB patients could reduce hippocampus mean dose (range) to 9.8 Gy (RBE) (6.1–11.8 Gy (RBE)) while keeping CTV_WB_ V_95%_ > 95 % (boost excluded). Using dose–response models, this sparing was estimated to lower the risk of late cognitive complications compared to various photon treatment techniques.

Gram et al., [25] created HS PBS proton plans with different hippocampus mean doses and concluded that for a mean dose of 9 Gy (RBE), plans for all included patients were deemed clinically acceptable. Estimated tumor control probability was relatively consistent between HS and non-HS plans, while long-term probability for inferior late cognitive complications (task efficiency, organization and memory) was significantly lower for HS plans. Plans were robustly optimized, and uncertainty criteria of 2%/2mm for both hippocampi and CTV were recommended. They showed that with 2%/2mm perturbations, it was possible to reduce the hippocampus dose compared to the more clinically used 3.5%/3mm while still maintaining clinically acceptable target coverage. Building on these results, we propose the advantages of reducing setup and range uncertainties to 2 mm and 2 %, respectively, in HS treatment. Recent publications have successfully demonstrated the clinical application of direct stopping power prediction using dual-energy CT, which would enable reduced range uncertainty from 3.5 % to 2 % [28], [29]. Also, Gram et al., [30] demonstrated residual set-up errors in the order of 1 mm using daily image-guided RT (IGRT) for pediatric CSI. From that perspective, 2 mm setup uncertainty was likely rather conservative and unlikely to occur with a careful IGRT protocol in which the cranial position is prioritized to be correct. Hence, 2%/2mm represents a relevant, if not conservative, estimation of clinically attainable uncertainty for these treatments.

The novelty of our study is the investigation of robustness and dosimetric plan verification of the treatment planning strategy developed by Gram et al., [25]. We showed that HS proton PBS plans are deliverable with high dosimetric accuracy and precision, demonstrating that proton HS treatment is dosimetrically feasible. Also, HS plans were relatively robust with respect to hippocampus mean dose (Fig. 2). However, although robustly optimized using 2%/2mm HS plans were less robust to these range and set-up uncertainties regarding target coverage and near maximum dose compared to non-HS plans. Observation of near-maximum doses of 120 % is limited to a few patients and specific uncertainty scenarios, considered worst-case situations. High-dose volumes are anticipated to smear out throughout the course of treatment due to random variations in patient setup. It is also worth noting that these high-dose volumes were small, and the prescribed dose is relatively low at 23.4 Gy (RBE). More realistic robust evaluations together with development of more robust treatment planning strategies are needed in the future.

Estimated decrease in risk of late cognitive complications must be balanced against potential increased risk of disease recurrence in peri-hippocampal regions when reducing dose to hippocampi. In this study we attempted to reduce the dose to only a small volume of the brain, approximately 1 % of the volume. It has been demonstrated that peri-hippocampal failures are uncommon in patients with non-metastatic MB [21], [27], which might suggest that HS PT could be a viable strategy to explore in a prospective trial for a suitable risk-group of MB patients.

A negative correlation was observed between pass rate and CTV_WB_ volume for both HS and non-HS plans (Table 1), suggesting an inferior dosimetric accuracy for larger fields. Significantly better agreement (p < 0.001) between measured and planned dose distributions was observed for larger measurement depths (approximately 10 cm) compared to shallower depths (approximately 5 cm) (Table 1 and Fig. 3). Same correlation was observed for both HS and non-HS plans and thus did not depend on the HS technique. Further, measured dose was systematically approximately 3 % higher in the entire field compared to planned dose for some of the patients for the shallower measurement depths. Limitations of the pencil beam (PB) algorithm in the current TPS are well known and large differences between dose distributions calculated using the PB algorithm and patient-specific QA measurements using the Matrixx detector have previously been demonstrated [31]. It has also been shown that the agreement between measured and calculated dose distributions depend on measurement depth within the spread-out Bragg peak [31]. Hence, the deviation between measurements and calculations observed in this study is likely due to beam modelling limitations of the PB algorithm. This discrepancy is larger for depths around 5 cm compared to 10 cm. Sharp dose gradients in the hippocampus region are delivered with a high precision as demonstrated in the high-resolution measurements.

Measurements were performed in 2D planes at a limited number of depths in the hippocampus region in a homogeneous water phantom using a detector with 7.6/3.8 mm resolution. Performing measurements at a greater number of depths, particularly immediately before and after the hippocampus region, would have been of interest. A more time-efficient way would be to measure the whole treatment plan at once, preferably in the patient geometry using an anthropomorphic phantom and employing detectors with higher resolution such as film or gel dosimetry. However, this is challenging due to the linear energy transfer (LET) dependency of both film and gel dosimeters, which might compromise measurement accuracy [32], [33]. Another limiting factor is that the plans were calculated using a PB algorithm. Monte Carlo would have resulted in a better dose calculation accuracy and hence probably better agreement between measured and calculated dose distributions. Also, all plans were calculated assuming a fixed RBE of 1.1. There is an increasing concern that the risk of normal tissue injuries may be underestimated using an RBE of 1.1 in dose calculation [34], [35], [36], [37]. Since LET and hence RBE is higher at the end of the protons range [38], [39], variable RBE-weighted dose to hippocampi and surrounding region could be higher compared to predictions using an RBE of 1.1 for HS plans and this should be further evaluated.

In conclusion, it was possible to reduce the dose to hippocampus using hippocampal-sparing treatment plans with very steep dose gradients. Measurement results were comparable to non-hippocampal-sparing plans and agreed well with the planned dose distribution in the hippocampus region despite steep dose gradients. Plans were relatively robust with respect to hippocampus mean dose, however more robust treatment planning strategies regarding target coverage and maximum dose should be developed.

## CRediT authorship contribution statement

**Anneli Edvardsson:** Conceptualization, Formal analysis, Investigation, Methodology, Visualization, Writing – original draft. **Jenny Gorgisyan:** Conceptualization, Investigation, Methodology, Writing – review & editing. **Karin M. Andersson:** Investigation, Methodology, Writing – review & editing. **Christina Vallhagen Dahlgren:** Investigation, Methodology, Writing – review & editing. **Alexandru Dasu:** Resources, Methodology, Writing – review & editing. **Daniel Gram:** Methodology, Writing – review & editing. **Thomas Björk-Eriksson:** Conceptualization, Writing – review & editing, Supervision, Funding acquisition. **Per Munck af Rosenschöld:** Conceptualization, Methodology, Writing – review & editing, Supervision, Funding acquisition.

## Declaration of competing interest

The authors declare the following financial interests/personal relationships which may be considered as potential competing interests: Per Munck af Rosenschöld: Research Agreement: Accuray Inc, US.
