# Supplementary material for: Robustness and dosimetric verification of hippocampal-sparing craniospinal pencil beam scanning proton plans for pediatric medulloblastoma
Source: Phys Imaging Radiat Oncol. 2024 Feb 15;29:100555. doi: 10.1016/j.phro.2024.100555 (PMC10891325; doi:10.1016/j.phro.2024.100555)
Supplement: Supplementary data 1 [file mmc1.docx]

## Supplementary material

**Supplementary table 1** Characteristics of the treatment given to the 15 patients included in the study

|  | Number of patients | Relative number of patients (%) |
| --- | --- | --- |
| Sex |  |  |
| Male | 8 | 53 |
| Female | 7 | 47 |
| Risk group |  |  |
| Standard | 11 | 73 |
| High  Prescribed dose CSI/boost/total [Gy (RBE)] | 4 | 27 |
| 23.4/30.6/54.0 | 11 | 73 |
| 35.2/19.8/55.0 | 2 | 13 |
| 36.0/18/54.0 | 2 | 13 |
| Boost volume |  |  |
| Tumor bed | 14 | 93 |
| Posterior fossa | 1 | 7 |
|  | Median | Range |
| Age (y) | 9 | 4-13 |
| Volume (cm^3^) |  |  |
| CTV_WB_ | 1416 | 1326-1759 |
| CTV_boost_ | 66 | 32-300 |
| Hippocampus | 3.3 | 1.4-7.0 |
